# Old age is perceived to begin later: cross-European differences and the role of macro-level factors for the historical change in the perceived onset of old age

**DOI:** 10.1093/geronb/gbag083

**Published:** 2026-05-12

**Authors:** Markus Wettstein, Rinseo Park, Moritz Hess, Jana Mäcken, Anna Wanka, Susanne Wurm, Nilam Ram, Denis Gerstorf

**Affiliations:** Humboldt-Universität zu Berlin, Department of Psychology, Berlin, Germany; Department of Prevention Research and Social Medicine, Institute for Community Medicine, University Medicine Greifswald, Greifswald, Germany; Departments of Communication and Psychology, Stanford University, Stanford, California, United States; Faculty of Social Sciences, Niederrhein University of Applied Sciences, Krefeld, Germany; Nortal AG, Berlin, Germany; Department of Educational Sciences, Goethe University Frankfurt, Frankfurt am Main, Germany; Department of Prevention Research and Social Medicine, Institute for Community Medicine, University Medicine Greifswald, Greifswald, Germany; Departments of Communication and Psychology, Stanford University, Stanford, California, United States; Socio–Economic Panel Study (SOEP), German Institute for Economic Research, DIW Berlin, Berlin, Germany; Humboldt-Universität zu Berlin, Department of Psychology, Berlin, Germany; Socio–Economic Panel Study (SOEP), German Institute for Economic Research, DIW Berlin, Berlin, Germany; (Psychological Sciences Section)

**Keywords:** Cohort effect, Views on aging, Historical change, Retirement age, Healthy life expectancy

## Abstract

**Objectives:**

The age at which people consider old age to begin (i.e., the perceived onset of old age) might have changed over the past decades due to changes in macro-level factors, such as statutory retirement age, older adults’ employment rate, and healthy life expectancy.

**Methods:**

We applied multilevel models to data from the European Social Survey (*n *= 55,721) to examine how change in perceived onset of old age—“At what age do men/women reach old age?”—over 12 years, measured in independent samples in 2006 and 2018 across 17 European countries, was related to macro-level changes in employment rate among those aged 55–64 years, gender-specific statutory retirement ages, and healthy life expectancy at age 65.

**Results:**

Results suggest that, on average, the perceived onset of old age was 3.7 years later in 2018 than in 2006. The perceived onset of old age in 2006 was later in countries with a higher healthy life expectancy in 2006. Increases in the perceived onset of old age between 2006 and 2018 were larger in countries with higher rises in men’s retirement age; increases were smaller in countries with greater gains in healthy life expectancy and with higher rises in older adults’ employment rates and in women’s retirement age between 2006 and 2018. Men perceived women to enter old age earlier than men.

**Discussion:**

Our findings suggest that theoretical frameworks of perceptions on old age need to consider the role of macro-level policies with regard to societal health, retirement, and age-related employment structure.

Most people have a concept of when “old age” begins, which we refer to here as “perceived onset of old age” (Barrett & Von Rohr, 2008; Wettstein et al., 2024, 2025). There are considerable interindividual differences in the perceived onset of old age. These are shaped both by micro-level factors that vary across individuals and by macro-level factors that vary across countries (Ayalon et al., 2014). So far, research has mostly focused on micro-level factors, such as age, education, or gender (e.g., Ayalon et al., 2014; Kessler & Warner, 2023; Wettstein et al., 2024, 2025). The perceived onset of old age has also been found to have changed across historical time (Augustyński & Jurek, 2021; Billari et al., 2021; Ennis et al., 2025; Wettstein et al., 2024), with later-born cohorts reporting a later perceived onset of old age compared to earlier-born cohorts. In addition, the perceived onset of old age and its historical change differ remarkably across cultures and nations (Augustyński & Jurek, 2021; Ayalon et al., 2014; Frąckowiak et al., 2020; Jurek, 2021; Swift et al., 2018). Against this backdrop, the aim of the present study is to investigate trends in the perceived onset of old age between 2006 and 2018 from a cross-national perspective by using data from the European Social Survey (ESS) (Fitzgerald & Jowell, 2010; Jowell et al., 2007), comprising 17 nations.

The HIDECO model (Historical changes in Developmental Contexts; Drewelies et al., 2018), which we use as a heuristic theoretical framework that guides our research questions, highlights health, occupation, and culture (operationalized as country-level differences in our study) as key developmental contexts shaping historical change in individual development. Based on this theoretical framework, we investigate three specific macro-level factors—as well as their historical change over time—that likely contribute to individuals’ perceived onset of old age: older adults’ employment rate, a country’s statutory retirement age of men and women, and healthy life expectancy at age 65. We also explore the role of gender in the perceived onset of old age and change therein, considering both the gender of targets (i.e., when men vs women reach old age) and of raters (i.e., when male vs female raters believe that old age begins).

## Historical change in perceived onset of old age

Different theoretical approaches support the assumption that the perceived onset of old age has increased over time. According to technophysio evolution theory (Fogel & Costa, 1997), advances in technology have “greatly improved the robustness and capacity of vital organ systems” (p. 49) and promoted longevity, which could potentially result in a later perceived onset of old age across historical time. Cowgill’s (1974) modernization theory postulates that modernization processes rather undermine the status of older adults across historical time and the consequence could be the same: In order to distance themselves from the group of older adults and from older adults’ low status, people might set the perceived onset of old age increasingly later as a means of “age-group dissociation” (Weiss & Kornadt, 2018). Furthermore, the theory of sociological institutionalism proposes that societal institutions (e.g., the welfare state) strongly influence not only individual behavior but also norms and values (e.g., the idea when somebody is old) (Glynn & D’Aunno et al., 2023). Against the background of a policy shift toward later retirement (Stiemke et al., 2025) and active aging (Marzo et al., 2023), sociological institutionalism would also assume a later perceived onset of old age over historical time.

Empirical studies do indeed support the hypothesis of a later perceived onset of old age across historical time, as historical trends toward a “postponement” of old age have been shown to amount to about 3 years among older adults in the United States who were born approximately 20 years apart (Ennis et al., 2025) and to even more than 5 years on average across European countries between 2008 and 2018 (Augustyński & Jurek, 2021).

## Macro-level factors and historical change in perceived onset of old age

On the macro-level, the health component of the HIDECO model corresponds to indicators such as healthy life expectancy, which varies considerably across countries (European Union, n.d.) and over historical time due to medical and health advances. Perceptions of when old age begins may partly depend on this factor; in countries with high healthy life expectancy, older adults may be seen—and see themselves—as “old” later.

Health and healthy life expectancy also vary markedly between countries (European Union, n.d.; Gimeno et al., 2024). As individuals might only be perceived as “old” when their health is compromised, given the common phenomenon that disease is often attributed to age (Levy et al., 2009), in countries where individuals stay healthy for a longer time across their lifespan, the average perceived onset of old age might be later. Empirically, findings indeed suggest that people in countries with higher healthy life expectancy at age 60 report later perceived onsets of old age (Jurek, 2021), but existing evidence is scarce.

Statutory retirement age and employment rates of older adults, representing the “occupation” component of the HIDECO model, may be additional macro-level factors linked to the perceived onset of old age. As Maung (2021) notes, the common idea that “65 marks the beginning of older adulthood” partly stems from this being the traditional pension age. Younger adults, in particular, seem to anchor their sense of when old age begins to statutory retirement ages, which in many countries occur in the early to mid-sixties (e.g., in Germany; Demografieportal, 2025; Rupprecht et al., 2025; Wurm et al., 2025).^1^

Empirical findings regarding the role of retirement age for the perceived onset of old age are mixed: Jurek (2021) observed a later average perceived onset of old age among countries with a later retirement age in two cross-national studies, but not in a third study. Augustyński and Jurek (2021) found a link between women’s retirement age and perceived old age in 2008, but not in 2018. Men’s retirement age was not significantly associated with the perceived onset of old age in 2008 and 2018. Neither men’s nor women’s retirement ages were significantly associated with historical change in the perceived onset of old age.

Another indicator of the occupation component in the HIDECO model is older adults’ employment rate. In countries with higher employment rates of older adults, age stereotypes might be more positive and emphasize “active” or “productive” aging, leading to a later perceived onset of old age. In contrast, in countries where older adults are excluded from the labor market, old age may be seen as beginning earlier. Empirical evidence indeed shows that increases in labor force participation among those aged 75+ are linked to later perceived onsets of old age (Augustyński & Jurek, 2021), although such effects were only partially corroborated across datasets (Jurek, 2021).

To summarize, we include in our analyses several macro-level indicators that represent the HIDECO model components. Specifically, the model components “health” and “occupation” will be represented by the healthy life expectancy of older adults, the statutory retirement age, and the employment rate of older adults. While there are a few studies that linked these macro-level indicators with the perceived onset of old age, most of them did not consider the role of historical change in these indicators and how it relates to historical change in the perceived onset of old age.

## The role of gender in perceived onset of old age

Gender is another component of the HIDECO model, which needs to be considered from a micro-level, interindividual differences perspective and which, so far, has rarely been investigated in combination with macro-level factors such as the ones described above. Female *raters* answering the question “When does old age begin?” typically set the beginning of old age later than do male raters (Ayalon et al., 2014; Barrett & Von Rohr, 2008; Chopik et al., 2018; Drevenstedt, 1976; Frąckowiak et al., 2020; Toothman & Barrett, 2011). This is presumably due to two factors: first, because women live longer than men (Eurostat, 2025, March). Second, age stereotypes tend to be more negative when directed at older women than at older men, a phenomenon known as the “double standard of aging” (Sontag, 1982). This form of “gendered ageism” (Rochon et al., 2021) may create stronger pressure on women to distance themselves from old age, so that women may be more likely than men to make use of “age-group dissociation” processes (Weiss & Kornadt, 2018) by setting the onset of old age later (and thus further away from their chronological age) than men.

As *targets* of the question “At what age do men/women reach old age?” women are perceived to enter old age earlier than men, particularly when rated by men (Barrett & Von Rohr, 2008; Billari et al., 2021; Drevenstedt, 1976; Toothman & Barrett, 2011). This gender disparity is quite universal across European countries (Billari et al., 2021) and across historical time. Women seem to be aware of that gender discrepancy, and they might “internalize” it, as they are more likely than men to believe that society considers them as old (Bordone et al., 2020).

## The present study

We compare the perceived onset of old age across 17 European countries and investigate how it changed, based on independent samples that were assessed in 2006 versus 2018. We examine how macro-level factors (older adults’ employment rate, statutory retirement age, and healthy life expectancy at age 65), as well as their historical changes, are related to country-level differences in the perceived onset of old age and its historical change. In addition, we take gender as a micro-level, interindividual difference factor into account. Men and women differ not only in how they perceive the onset of old age (i.e., gender *rater* differences) but also in when they are perceived to enter into old age (i.e., gender *target* differences). To our knowledge, this is the first study that investigates the role of this specific set of macro-level factors—including their historical change—for historical change in the perceived onset of old age by additionally taking the “twofold” role of gender (as target and as rater) into account.

Our hypotheses are as follows:

1. In line with prior research (Augustyński & Jurek, 2021; Jurek, 2021), we expect that baseline perceived onset of old age (in 2006) is later in countries with higher macro-level indicators, namely, older adults’ employment rates, men’s and women’s statutory retirement ages, and healthy life expectancy.
2. We expect that the perceived onset of old age has shifted toward older ages between 2006 and 2018, in line with prior research (Augustyński & Jurek, 2021). We additionally expect the historical shift toward a later perceived onset of old age to be more pronounced in countries that also had higher increases in macro-level indicators, namely older adults’ employment rates, men’s and women’s statutory retirement ages, and healthy life expectancy.
3. We assume that gender plays an important role in the perceived onset of old age, with female raters generally reporting a later onset of old age than male raters and female targets being perceived as entering old age earlier than male targets.

## Method

### Data source

Data were drawn from the ESS (Fitzgerald & Jowell, 2010), a biennial multi-country survey across Europe that started in 2001, supplemented with publicly available data obtained from the OECD Data Explorer (Organisation for Economic Co-operation and Development; OECD, n.d.; OECD, 2007, 2018), the Social Security Administration (2006, 2018), and the Eurostat database. European Social Survey data consist of probability-based cross-sectional samples, with participants randomly drawn in each wave from individuals aged 15 and older, living in private households. Data collection was based on face-to-face personal interviews using questionnaires from ESS. The present study focuses on two waves, 2006 (Round 3; European Social Survey European Research Infrastructure, 2018a, 2018b) and 2018 (Round 9; European Social Survey European Research Infrastructure, 2023a, 2023b), when the survey included questions about participants’ perceived onset of old age (Billari et al., 2021). The final sample across the two assessment years included *N *= 55,721 individual ratings from 17 countries.

### Measures

#### Micro-level variables

**Onset of old age.** Participants were asked, “At what age, approximately, would you say [women/men] reach old age?” Based on a gender-specific split-ballot design, half of the sample answered questions for “men” as targets (*n *= 27,782) and the other half answered for “women” as targets (*n *= 27,939); gender target was, thus, a between-person design factor that differentiated the outcome variable in separate analyses for all/men/women targets.

**Year of assessment** was coded as a binary variable with 0 = 2006 (Round 3) and 1 = 2018 (Round 9). In the final sample, 28,999 individual ratings were collected in 2006, and 26,722 individual ratings were collected in 2018.

**Gender** of the respondents was coded as a binary variable (0 = male, 1 = female).

**Age** was assessed as years of chronological age at the time of the survey. The measure was mean-centered using the weighted mean (*M *= 47.1) across the two observations and rescaled such that one unit corresponds to 10 years.

**Education** was assessed as years of completed full-time education at the time of the survey. The measure was mean-centered using the weighted mean (*M *= 12.6) across the two observations and rescaled such that one unit corresponds to 10 years.

#### Macro-level variables

For each macro-level variable, values from 2006 were treated as baseline, and change scores were calculated as the score in 2018 minus the score in 2006. For the baseline variables, one unit corresponds to 10 years, and for the change variables, one unit corresponds to 1 year.

**Employment rate.** Older adults’ (55–64 years) employment rates (not differentiated by gender) in each country for 2006 and 2018 were obtained from the OECD database (OECD, n.d.). For the baseline measure, the employment rate was mean-centered using the weighted mean (*M *= 49.2%) and rescaled such that one unit corresponds to 10%. Change scores were calculated as the employment rate in 2018 minus the employment rate in 2006. For the change measure, one unit corresponds to 1%.

**Retirement age for men and women** in each country for 2006 and 2018 was obtained from the OECD database (OECD, 2007, 2018) and for Estonia, Hungary, and Slovenia from the Social Security Administration (2006, 2018). For the baseline measure, retirement age was mean-centered using the weighted mean, *M *= 65.1 for women and *M *= 67.3 for men, then rescaled such that one unit corresponds to 10 years. For the change measure, one unit corresponds to 1 year.

**Healthy life expectancy** (i.e., healthy life years in absolute value at age 65; not differentiated by gender) in each country for 2006 and 2018 was obtained from the Eurostat database (Eurostat, n.d.). For the baseline measure, healthy life expectancy was mean-centered using the weighted mean (*M *= 8.4) and rescaled such that one unit corresponds to 10 years. For the change measure, one unit corresponds to 1 year.

### Data analysis

To examine how individuals’ perceived onset of old age changed across Europe between 2006 and 2018, we used multilevel models that accommodate the nested structure of the data (i.e., individual ratings within each country). Specifically, individual ratings of the perceived onset of old age were modeled as:


(1) $$\mathrm{OnsetO}A_{ij}=\beta_{0j}+\beta_{1j}\left(\mathrm{Yea}r_{ij}\right)+\beta_{2j}\left(\mathrm{FemaleRate}r_{ij}\right)+\beta_{3j}\left(\mathrm{Yea}r_{ij}\times \mathrm{FemaleRate}r_{ij}\right)+\beta_{4j}\left(Age_{ij}\right)+\beta_{5j}\left(\mathrm{Educatio}n_{ij}\right)+e_{ij}$$

where the ratings of *onset of old age* made by individual *i* in country *j*, *OnsetOA_ij_*, obtained across two assessment years, are modeled for each country *j* as a function of *person-level variables*, including the *year of assessment* (0 = 2006, 1 = 2018), *gender of the rater* (0 = male, 1 = female), and their interaction. *Age* and *years of education* of the rater were included as covariates, as these micro-level variables are systematically associated with perceived onset of old age (Billari et al., 2021; Chopik et al., 2018; Rupprecht et al., 2025; Wettstein et al., 2024; Wurm et al., 2025). Residual unexplained individual differences *e_ij_* are assumed to follow a normal distribution *N*(0, *σ_e_*^2^).

The intercepts and regression coefficients in *Equation 1* are simultaneously modeled as functions of *country-level* indicators (see *Equations* 2.1–2.4), which include country-specific baseline (*Employ_j_*, *MenRetire_j_*, *WomenRetire_j_*, *Healthy_j_*) and change measures (*ChangeEmploy_j_*, *ChangeMenRetire_j_*, *ChangeWomenRetire_j_*, *ChangeHealthy_j_*).


(2.1) $$\beta_{0j}=\gamma_{00}+\gamma_{01}\left(\mathrm{Emplo}y_{j}\right)+\gamma_{02}\left(\mathrm{MenRetir}e_{j}\right)+\gamma_{03}\left(\mathrm{WomenRetir}e_{j}\right)+\gamma_{04}\left(\mathrm{Health}y_{j}\right)+u_{0j}$$

(2.2) $$\begin{array}{l} \beta_{1j}=\gamma_{10}+\gamma_{11}\left(\mathrm{Emplo}y_{j}\right)+\gamma_{12}\left(\mathrm{MenRetir}e_{j}\right)+\gamma_{13}\left(\mathrm{WomenRetir}e_{j}\right)+\gamma_{14}\left(\mathrm{Health}y_{j}\right)+\gamma_{15}(\mathrm{ChangeEmplo}y_{j})+\gamma_{16}(\mathrm{ChangeMenRetir}e_{j})+\gamma_{17}(\mathrm{ChangeWomenRetir}e_{j})+\gamma_{18}(\mathrm{ChangeHealth}y_{j})+\gamma_{19}\left(\mathrm{Emplo}y_{j}\times \mathrm{ChangeEmplo}y_{j}\right)+\gamma_{110}(\mathrm{MenRetir}e_{j}\times C\mathrm{hangeMenRetir}e_{j})+\gamma_{111}\left(\mathrm{WomenRetir}e_{j}\times \mathrm{ChangeWomenRetir}e_{j}\right)+\gamma_{112}(\mathrm{Health}y_{j}\times C\mathrm{hangeHealth}y_{j}) \end{array}$$

(2.3) $$\beta_{2j}=\gamma_{20}+\gamma_{21}\left(\mathrm{Emplo}y_{j}\right)+\gamma_{22}\left(\mathrm{MenRetir}e_{j}\right)+\gamma_{23}\left(\mathrm{WomenRetir}e_{j}\right)+\gamma_{24}\left(\mathrm{Health}y_{j}\right)$$

(2.4) $$\begin{array}{l} \beta_{3j}=\gamma_{30}+\gamma_{31}\left(\mathrm{Emplo}y_{j}\right)+\gamma_{32}\left(\mathrm{MenRetir}e_{j}\right)+\gamma_{33}\left(\mathrm{WomenRetir}e_{j}\right)+\gamma_{34}\left(\mathrm{Health}y_{j}\right)+\gamma_{35}(\mathrm{ChangeEmplo}y_{j})+\gamma_{36}(\mathrm{ChangeMenRetir}e_{j})+\gamma_{37}(\mathrm{ChangeWomenRetir}e_{j})+\gamma_{38}(\mathrm{ChangeHealth}y_{j})+\gamma_{39}\left(\mathrm{Emplo}y_{j}\times \mathrm{ChangeEmplo}y_{j}\right)+\gamma_{310}(\mathrm{MenRetir}e_{j}\times C\mathrm{hangeMenRetir}e_{j})+\gamma_{311}\left(\mathrm{WomenRetir}e_{j}\times \mathrm{ChangeWomenRetir}e_{j}\right)+\gamma_{312}(\mathrm{Health}y_{j}\times C\mathrm{hangeHealth}y_{j}) \end{array}$$

(2.5) $$\beta_{4j}=\gamma_{40}$$

(2.6) $$\beta_{5j}=\gamma_{50}$$

In addition to fixed effects for all the country- and person-level predictors, the model includes random effects on intercepts *u_0j_* that allow country-level baseline ratings in 2006 to vary; *γ_00_* is the expected perceived onset of old age in 2006 in the average country, and *γ_01_* is the difference in perceived onset of old age in 2006 for a unit difference (=10%) in employment rate. Additional details about these *Equations* (2.1–2.6) are given in the Supplementary Material.^2^

Based on the split-ballot design, the models were fitted separately for (M1) the full sample, (M2) the men target group (i.e., male and female respondents receiving the item “at what age *men* reach old age”), and (M3) the women target group (i.e., male and female respondents receiving the item “at what age *women* reach old age”). All three models were fitted with survey weights^3^ provided in ESS data using the R “lme4” (version 1.1-35) package, based on the assumption that data are missing at random (Little & Rubin, 2002).^4^ Statistical significance was evaluated with alpha = .05.

## Results

**Figure 1:**
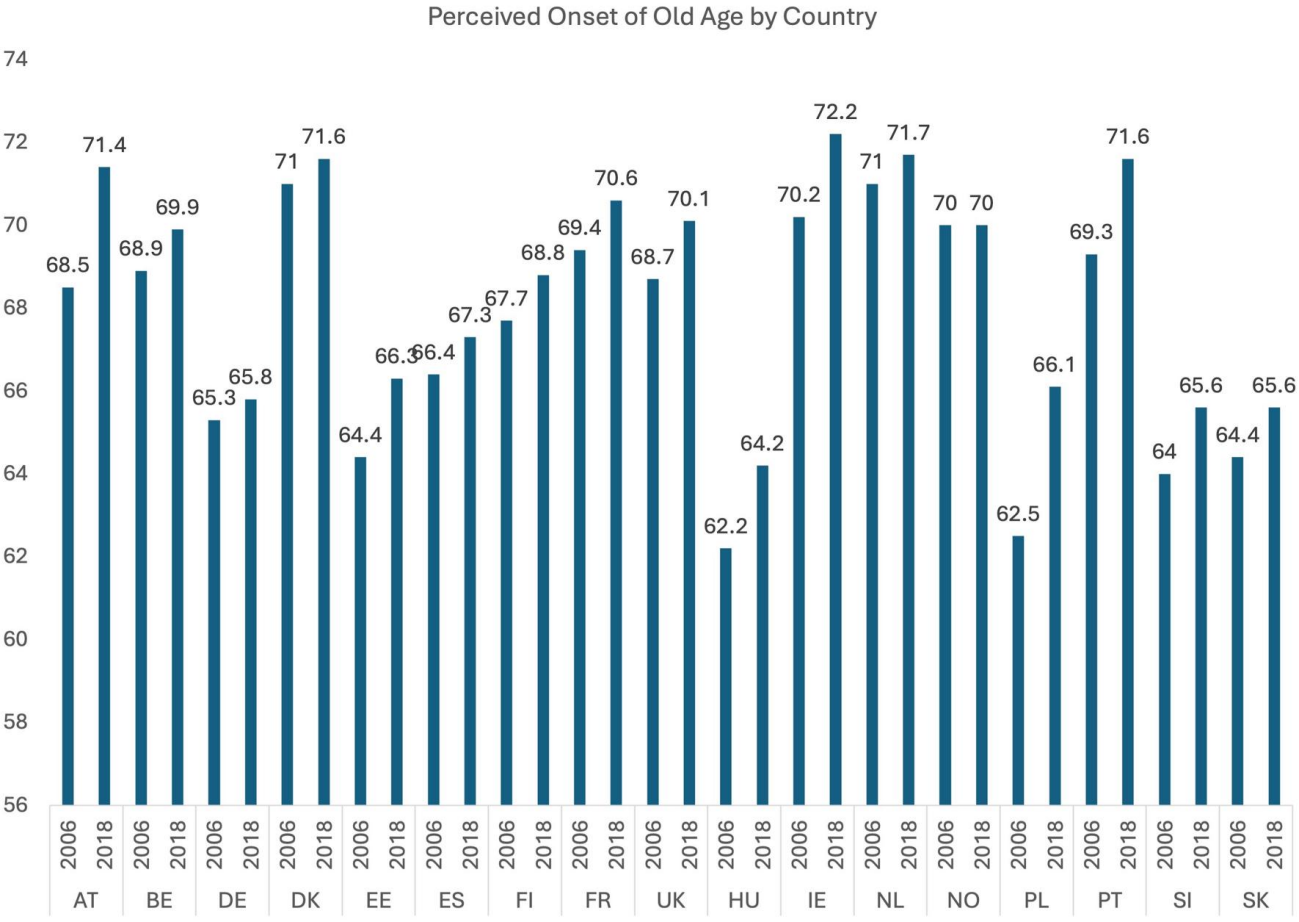
Historical change in perceived onset of old age by country. AT = Austria; BE = Belgium; DE = Germany; DK = Denmark; EE = Estonia; ES = Spain; FI = Finland; FR = France; UK = United Kingdom; HU = Hungary; IE = Ireland; NL = Netherlands; NO = Norway; PL = Poland; PT = Portugal; SI = Slovenia; SK = Slovakia.

Descriptive statistics for the macro-level indicators across countries are provided in Table S1. Table 1 shows the descriptive statistics for socio-demographic indicators and perceived onset of old age (see Table S2 for descriptive statistics with post-stratification weights). The descriptive data from Table 1 suggest an increase in the perceived onset of old age between 2006 and 2018, with considerable cross-country variation in the extent of historical change, which is illustrated in Figure 1.

**Table 1:** Country-level descriptive statistics.

Country	Year	Female ratio	Mean age	Min. age	Max. age	Education	Perceived onset of old age		
							All targets	Men targets	Women targets
**Austria**	2006	0.53	44.3	15	93	12.5	68.5	68.6	68.4
	2018	0.54	51.1	15	90	12.6	71.4	71.7	71.1
**Belgium**	2006	0.53	45.5	14	95	12.1	68.9	69.2	68.5
	2018	0.51	47.4	15	90	13.7	69.9	70.0	69.8
**Germany**	2006	0.50	47.4	15	96	13.2	65.3	66.1	64.5
	2018	0.48	48.6	15	90	14.4	65.8	66.0	65.6
**Denmark**	2006	0.50	48.2	16	95	13.2	71.0	71.1	70.8
	2018	0.46	49.2	15	90	13.7	71.6	71.8	71.5
**Estonia**	2006	0.57	46.9	15	100	12.3	64.4	64.7	64.1
	2018	0.56	49.7	15	90	13.4	66.3	66.0	66.6
**Spain**	2006	0.51	45.6	15	97	11.7	66.4	66.7	66.1
	2018	0.50	47.7	15	90	13.3	67.3	67.3	67.3
**Finland**	2006	0.52	47.5	15	97	12.6	67.7	67.4	68.0
	2018	0.52	50.2	15	90	14.1	68.8	68.5	69.2
**France**	2006	0.53	47.3	15	95	12.5	69.4	69.8	69.1
	2018	0.55	51.2	15	90	13.2	70.6	70.8	70.4
**United Kingdom**	2006	0.54	48.8	15	97	13.5	68.7	69.1	68.2
	2018	0.55	52.1	15	90	14.3	70.1	69.9	70.4
**Hungary**	2006	0.59	50.8	15	93	11.7	62.2	63.2	61.2
	2018	0.57	51.0	16	90	12.3	64.2	64.7	63.6
**Ireland**	2006	0.63	46.0	15	98	12.8	70.2	70.3	70.1
	2018	0.54	51.9	15	90	14.6	72.2	71.9	72.4
**Netherlands**	2006	0.54	48.4	15	94	13.2	71.0	71.1	71.0
	2018	0.50	47.9	15	90	14.3	71.7	71.5	72.0
**Norway**	2006	0.49	45.4	15	101	13.4	70.0	70.2	69.7
	2018	0.45	46.6	15	90	14.1	70.0	70.1	69.9
**Poland**	2006	0.53	43.5	15	86	11.5	62.5	63.3	61.8
	2018	0.53	47.6	15	87	12.8	66.1	66.8	65.4
**Portugal**	2006	0.60	50.9	15	94	7.2	69.3	69.5	69.1
	2018	0.58	51.4	15	90	10.6	71.6	71.6	71.6
**Slovenia**	2006	0.54	45.8	15	96	11.7	64.0	64.0	64.0
	2018	0.53	49.0	15	90	12.8	65.6	65.9	65.3
**Slovakia**	2006	0.55	43.2	15	90	12.5	64.4	65.4	63.5
	2018	0.54	55.0	16	90	12.7	65.6	66.5	64.7

### Historical change in perceived onset of old age

Table 2 shows the results from multilevel models for the perceived onset of old age. As the gender of the raters was dummy-coded, with 0 corresponding to the reference group of male raters, the main effects refer to male raters, whereas the interaction terms with “female rater” indicate differences between male and female raters. In this section, we first interpret results from the full sample (“All Targets”) and then summarize the findings from models for “Men Targets” and “Women Targets.”

**Table 2:** Results from multilevel models examining relations between person- and country-level factors and individuals’ perceived onset of old age.

Effects	With covariates combined		
	All targets	Men targets	Women targets
***Fixed effects***			
**Intercept (*γ_00_*)**	66.64*** (1.25)	67.61*** (1.17)	65.61*** (1.37)
Employ (*γ_01_*)	0.21 (0.61)	0.46 (0.58)	−0.00 (0.67)
MenRetire (*γ_02_*)	−1.32 (4.29)	−1.76 (4.01)	−0.92 (4.70)
WomenRetire (*γ_03_*)	3.42 (2.75)	3.49 (2.60)	3.06 (3.03)
**Healthy (*γ_04_*)**	5.86* (2.42)	5.09* (2.32)	6.67* (2.69)
**Year (*γ_10_*)**	3.70*** (0.91)	4.06** (1.27)	3.35** (1.29)
× Employ (*γ_11_*)	−0.33 (0.40)	−0.54 (0.56)	−0.24 (0.58)
× MenRetire (*γ_12_*)	5.58* (2.74)	8.48* (3.80)	2.62 (3.89)
× WomenRetire (*γ_13_*)	−3.01* (1.23)	−2.42 (1.71)	−3.40 (1.75)
× Healthy (*γ_14_*)	−1.83 (1.30)	−0.92 (1.82)	−2.81 (1.83)
× ChangeEmploy (*γ_15_*)	−0.14*** (0.04)	−0.17** (0.06)	−0.11 (0.06)
× ChangeMenRetire (*γ_16_*)	2.05* (0.87)	1.13 (1.20)	2.89* (1.22)
× ChangeWomenRetire (*γ_17_*)	−0.59* (0.30)	−0.69 (0.41)	−0.45 (0.42)
× ChangeHealthy (*γ_18_*)	−0.23* (0.12)	−0.23 (0.17)	−0.26 (0.17)
× Employ × ChangeEmploy (*γ_19_*)	−0.04 (0.03)	−0.07 (0.04)	−0.00 (0.04)
× MenRetire × ChangeMenRetire (*γ_110_*)	1.92 (1.33)	0.15 (1.85)	3.61 (1.88)
× WomenRetire × ChangeWomenRetire (*γ_111_*)	−0.19 (0.41)	−0.46 (0.57)	0.16 (0.59)
× Healthy × ChangeHealthy (*γ_112_*)	−0.60 (0.57)	−1.21 (0.79)	0.10 (0.82)
**FemaleRater (*γ_20_*)**	2.09*** (0.20)	0.96*** (0.27)	3.25*** (0.28)
× Employ (*γ_21_*)	0.19 (0.12)	−0.39* (0.17)	0.75*** (0.18)
× MenRetire (*γ_22_*)	−0.23 (0.60)	0.27 (0.83)	−0.81 (0.85)
× WomenRetire (*γ_23_*)	−0.86 (0.46)	−0.86 (0.64)	−0.61 (0.65)
× Healthy (*γ_24_*)	0.50 (0.68)	1.42 (0.94)	−0.38 (0.97)
Year × FemaleRater (*γ_30_*)	−0.08 (1.05)	−0.17 (1.46)	0.00 (1.50)
× Employ (*γ_31_*)	0.80 (0.47)	1.05 (0.66)	0.59 (0.67)
× MenRetire (*γ_32_*)	4.59 (3.15)	3.02 (4.38)	6.50 (4.51)
× WomenRetire (*γ_33_*)	2.24 (1.45)	2.19 (2.02)	2.18 (2.07)
× Healthy (*γ_34_*)	−0.24 (1.58)	−2.67 (2.21)	2.06 (2.24)
× ChangeEmploy (*γ_35_*)	0.03 (0.05)	0.09 (0.07)	−0.02 (0.07)
× ChangeMenRetire (*γ_36_*)	−1.21 (0.98)	−0.49 (1.37)	−1.95 (1.38)
× ChangeWomenRetire (*γ_37_*)	0.71* (0.34)	0.43 (0.47)	0.97* (0.48)
× ChangeHealthy (*γ_38_*)	0.29* (0.14)	0.13 (0.19)	0.45* (0.19)
× Employ × ChangeEmploy (*γ_39_*)	−0.04 (0.04)	0.00 (0.05)	−0.08 (0.05)
× MenRetire × ChangeMenRetire (*γ_310_*)	−2.58 (1.50)	−1.76 (2.11)	−3.51 (2.12)
× WomenRetire × ChangeWomenRetire (*γ_311_*)	−0.04 (0.47)	−0.22 (0.65)	0.09 (0.68)
× Healthy × ChangeHealthy (*γ_312_*)	−1.01 (0.65)	0.21 (0.90)	−2.21* (0.92)
**Age (*γ_40_*)**	1.32*** (0.02)	1.29*** (0.03)	1.35*** (0.03)
**Education (*γ_50_*)**	2.11*** (0.08)	2.07*** (0.12)	2.14*** (0.12)
***Random effects***			
Std. Dev. Intercept (*σ_u0_*)	2.15	1.99	2.34
Residual Std. Dev. (*σ_e_*)	8.17	8.05	8.24
*Goodness-of-fit statistics*			
Log-likelihood (*df*)	−193,470 (40)	−106,204 (40)	−107,422 (40)
AIC	387,021	212,488	214,925
*Sample size*	55,721 (17)	27,782 (17)	27,939 (17)

*Note*. Statistically significant effects are printed in bold. AIC = Akaike information criterion. *N *= 55,721 from 17 countries. Survey weights applied in a combined way (see Table S3, “Combined”). Values in parentheses represent standard errors. Employ = employment rate of those aged 55-65 years; MenRetire = men's statutory retirement age; WomenRetire = women's statutory retirement age; Healthy = healthy life expectancy at age 65.

*** *p* < .001;

** *p* < .01;

* *p* < .05.

**Figure 2:**
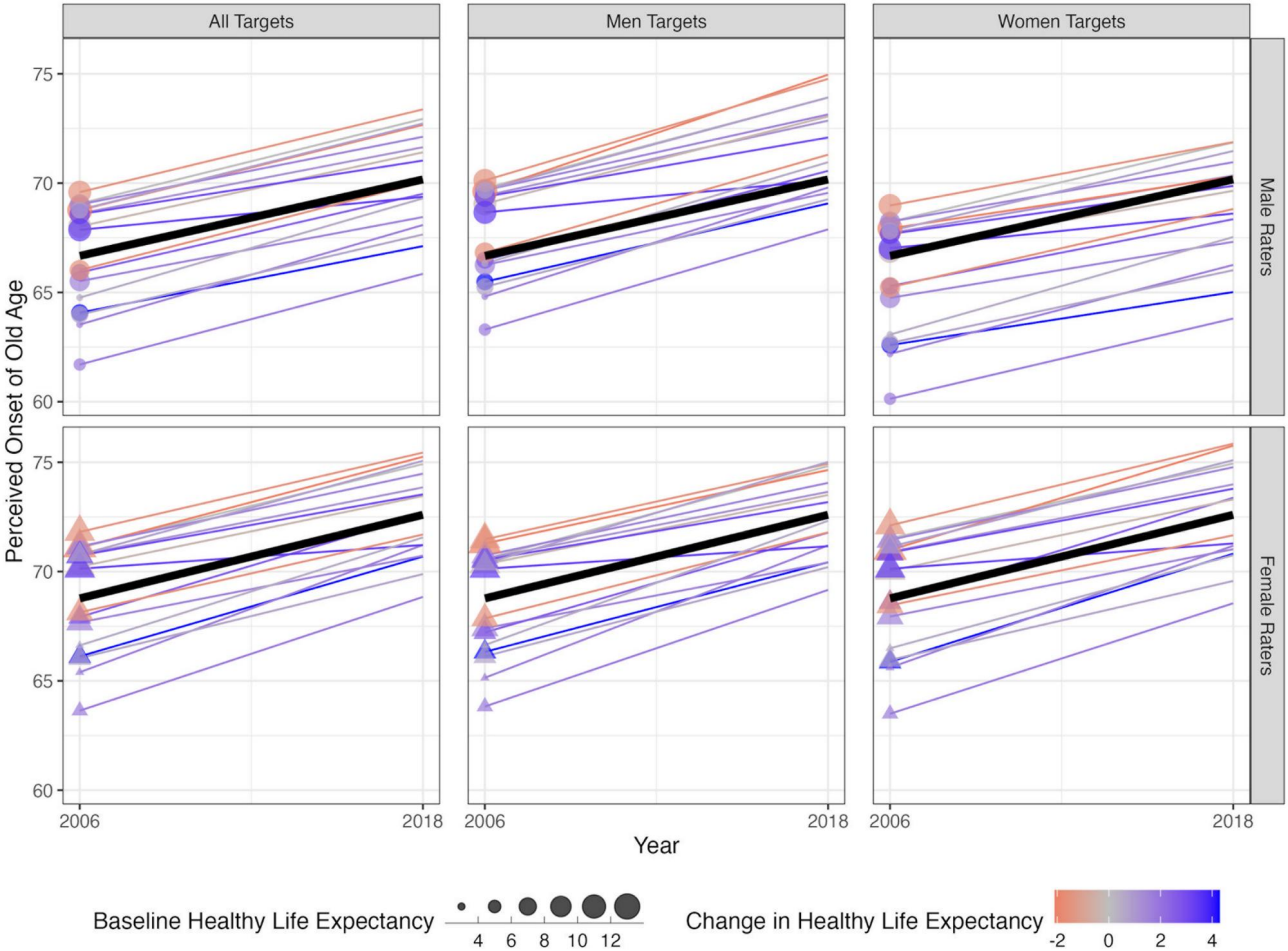
Model predicted historical change in perceived onset of old age for male and female raters in relation to country-level change in healthy life expectancy. Model predictions for male (circle) and female (triangle) raters in each country, given the initial level (2006) of and observed amount of change (2006–2018) in healthy life expectancy across two cohorts.

In line with our expectation of historical change in perceived onset of old age, the average perceived onset of old age was 3.7 years later in 2018 than in 2006 (see Figure 2). Specifically, the average onset from the perspective of male raters was 66.6 years in 2006 (*γ_00_* = 66.64, *p* < .001) and 3.7 years higher in 2018, amounting to 70.3 years in 2018 (*γ_10_* = 3.70, *p* < .001).

Also, as expected, country-level variables were related to both initial levels (2006) and changes between 2006 and 2018 in the perceived onset of old age. First, a higher baseline healthy life expectancy was related to later perceived onset of old age (*γ_04_* = 5.86, *p* = .03). Each additional year of baseline (2006) healthy life expectancy at age 65 was associated with 0.59 years higher perceived onset of old age (i.e., 1 unit = 10 years). Contrary to our hypotheses, neither differences in older adults’ employment rates nor differences in the retirement ages of men and women were significantly associated with differences in the perceived onset of old age (see *γ_01_*–*γ_03_*).

Regarding country-level changes in the perceived onset of old age between 2006 and 2018 (see *γ_11_*–*γ_112_*), the increase was steeper in countries with a higher male statutory retirement age in 2006 and—surprisingly—in countries with a lower female statutory retirement age in 2006. Specifically, in countries in which men’s statutory retirement age was higher by 1 year, the perceived onset of old age increased by 0.56 years (*γ_12_* = 5.58, *p* = .04), and perceived onset of old age increased more over 12 years in countries with a steepter increase in men's statutory retirement age, whereas in countries where women’s statutory retirement age was higher by 1 year, the perceived onset of old age decreased by 0.30 years (*γ_13_* = −3.01, *p* = .01). Contrary to our expectations, changes toward a later perceived onset of old age were less pronounced in countries that experienced greater increases in older adults’ employment rate, in the retirement age of women, and in healthy life expectancy between 2006 and 2018 (i.e., the change-on-change associations). Specifically, each additional percentage of increase in older adults’ employment rate (i.e., 1 unit = 1%) decreased the slope of perceived onset of old age by 0.14 (*γ_15_* = −0.14, *p* < .001); each additional year of increase in women’s retirement age and in healthy life expectancy (i.e., 1 unit = 1 year) decreased the slope by −0.59 and −0.23, respectively (*γ_17_* = −0.59, *p* = .046; *γ_18_* = −0.23, *p* = .049).

Person-level variables were also significantly associated with initial levels of the perceived onset of old age in 2006. Raters who were chronologically older by 1 year had a perceived onset that was on average 0.13 years later (*γ_40_* = 1.32, *p* < .001), and each additional year of education was associated with a 0.21-year-later perceived onset of old age (*γ_50_* = 2.11, *p* < .001).

### Perceived onset of old age of female and male raters

As expected in Hypothesis 3, the perceived onset of old age also differed in relation to the rater’s gender. In 2006, female raters reported a later perceived onset of old age than male raters (*γ_20_* = 2.09, *p* < .001; see Figure 2). Yet, this “gendered rater bias” was not significantly associated with any macro-level variables (see *γ_21_*–*γ_24_*). Moreover, female and male raters did not significantly differ in the extent of historical change of perceived onset of old age from 2006 to 2018 (*γ_30_* = −0.08, *p* = .94). However, there were significant moderation effects of women’s statutory retirement age and healthy life expectancy (*γ_37_* = 0.71, *p* = .03; *γ_38_* = 0.29, *p* = .03). Whereas for male raters, change in women’s statutory retirement age and change in healthy life expectancy were negatively related to change in perceived onset of old age, associations were around zero or positive for female raters. Healthy life expectancy, perceived onset of old age and historical change in both variables for each country by gender of the raters are shown in Figure S1.

### Perceived onset of old age of men versus women as targets

As shown in Table 2 and Figure 2, women were perceived to enter old age on average 2 years earlier than men (*γ_00_* = 67.61 for “men”; *γ_00_* = 65.61 for “women”). At the same time, the change in the onset of old age between 2006 and 2018 was less steep for women as targets than for men as targets (*γ_10_* = 4.06 for “men”; *γ_10_* = 3.35 for “women”). Moreover, the difference in when women, compared to men, are perceived to transition into old age is mostly caused by gender-specific perceptions of male raters. Specifically, for male raters, the average perceived onset of old age (*γ_00_*) of men is at 67.61 years, and the perceived onset of women is at 65.61 years, which corresponds to a difference of 2 years. For female raters, the average perceived onset of old age (*γ_00_ + γ_20_*) of men is at 68.57 years, and the average perceived onset of old age of women is at 68.86 years, a difference of only 0.29 years.

Additionally, the effects of country-level variables varied according to whether men or women were the target. Specifically, a later male retirement age was significantly associated with a later perceived onset of old age of men as targets (*γ_12_* = 8.48, *p* = .02*)*, but not of women as targets (*γ_12_ =* 2.62, *p* = .50). A greater change toward a higher employment rate of older adults was associated with a less steep historical increase of the perceived onset of old age of men as targets (*γ_15_ =* −0.17, *p* = .003), but not of women as targets (*γ_15_* = −0.11, *p* = .07). Moreover, among female raters, a change toward an older retirement age of women was associated with a greater change toward a later perceived onset of women as targets (*γ_37_* = 0.97, *p* = .04*)*, but not of men as targets (*γ_37_* = 0.43, *p* = .36). Similarly, for female raters, a change toward a longer healthy life expectancy at age 65 was associated with a change toward a later perceived onset of old age of women as targets (*γ_38_ =* 0.45, *p* = .02), but it was not associated with a change in the perceived onset of old age of men as targets (*γ_38_* = 0.13, *p* = .50). Healthy life expectancy, perceived onset of old age and historical change in both variables by gender of the target and by gender of raters for each country are shown in Figure S2.

## Discussion

We investigated how the perceived onset of old age changed between 2006 and 2018, using data from the ESS comprising 17 nations and 55,721 individuals. Using the HIDECO model (Drewelies et al., 2018) as a guiding heuristic theoretical framework that specifies determinants of historical change, we analyzed the role of macro-level factors (employment rate of older adults, mandatory retirement age of men, mandatory retirement age of women, healthy life expectancy of older adults) and their change for the 12-year historical trend in perceived onset of old age. Moreover, we examined the extent to which male and female raters differ in their perceived onset of old age and how macro-level factors relate to these perceptions and their 12-year change. Finally, we separately considered “perceived onset of old age for men as targets” versus “perceived onset of old age for women as targets,” and their associations with macro-level factors.

### Change in perceived onset of old age

Between 2006 and 2018, the perceived onset of old age increased by 3.70 years and was thus “postponed” from an average of 66.64 years in 2006 to the early seventies in 2018. This historical change toward a later perceived onset of old age confirms our hypothesis and is in line with other studies (e.g., Augustyński & Jurek, 2021, who also used ESS data). This change might reflect that contemporary life trajectories are more diverse, complex, and longer than they were in the past (Albertini & Vignoli, 2025). At the same time, one other study found the historical change to be less pronounced and limited to older adults only in a sample from the United States (Ennis et al., 2025), and yet another study observed a nonlinear and recently stagnating trend toward a later perceived onset of old age in a sample from Germany (Wettstein et al., 2024). Differences in the sample composition, particularly with regard to age and cultural background/country, may to some extent explain such divergent findings. Moreover, with the availability of only two measurement occasions at which the perceived onset of old age was assessed in the present study, we were not able to test potentially nonlinear temporal trends in the perceived onset of old age.

### The role of macro-level factors in the perceived onset of old age

As expected, older adults’ healthy life expectancy was significantly related to a later perceived onset of old age in 2006 (see also Jurek, 2021). In countries and societies with a longer healthy life expectancy at age 65, older adults stay healthy for a longer time and might, as a consequence, be perceived as younger and as transitioning into old age later than in countries where late-life healthy life expectancy is shorter.

In contrast to our expectations, the perceived onset of old age in 2006 was not significantly related to the statutory retirement ages of women and men or to older adults’ employment rates. The nonsignificant role of retirement age for perceived onset of old age is in line with another study (Ayalon et al., 2014), though not with others (Augustyński & Jurek, 2021; Jurek, 2021). The operationalization of retirement age might make an important difference for these discrepant findings. Alternative indicators, such as working life expectancy, which reveals some cross-nation variation across European countries, might be more strongly linked with the perceived onset of old age than statutory retirement age (Ophir, 2022).

Older adults’ employment rates were not significantly associated with the perceived onset of old age in 2006 as well. Maybe this information on the employment rate of older adults is not available to many individuals, so that across individuals of all ages and across all nations, this factor turned out to be not relevant.

### The role of macro-level factors for historical change in perceived onset of old age

We found that in countries with an older statutory retirement age for men and in countries where men’s retirement age was raised to a larger extent between 2006 and 2018, the change toward a later perceived onset of old age was more pronounced. This is in line with our expectation that retirement age might be an “anchor” for setting the onset of old age, although the initial perceived onset of old age was not significantly related to men’s retirement age. Unexpectedly, in countries with a later retirement age of women and in countries where women’s retirement age increased more between 2006 and 2018, historical change toward a later perceived onset of old age was less pronounced. However, as these counterintuitive associations were not replicated when women or men as targets were considered separately, the robustness of the associations is questionable, and the effects should not be overinterpreted.

Additional unexpected findings were that in countries with a stronger increase in older adults’ employment rate and in healthy life expectancy, the change toward a later perceived onset of old age was less pronounced. As the initial healthy life expectancy in 2006 was positively related to the perceived onset of old age in 2006, countries with a longer healthy life expectancy at age 65 in 2006 might have improved less between 2006 and 2018, so that the association of healthy life expectancy changes with change in perceived onset of old age could reflect regression toward the mean. However, this is a speculative explanation that requires further testing. Alternatively, an increase in healthy life expectancy might reduce individuals’ “fear of aging,” so that they feel less pressured to set a late onset of old age in order to distance themselves from the group of older adults. For the role of change in older adults’ employment rate, it is possible that a greater proportion of older adults in the labor market does not necessarily promote a later perceived onset of old age, and there might be confounding factors. For instance, in poorer countries with insufficient pension systems, people might be forced to stay in the workforce longer, and this involuntary late-life labor market participation might be associated with earlier, rather than later, perceived onsets of old age.

### Perceived onset of old age from a gender-based perspective

Our findings are in line with extant reports that female raters set the onset of old age later than male raters (Ayalon et al., 2014; Barrett & Von Rohr, 2008; Chopik et al., 2018; Drevenstedt, 1976; Frąckowiak et al., 2020; Toothman & Barrett, 2011), and women were perceived to enter old age earlier than men (Barrett & Von Rohr, 2008; Drevenstedt, 1976; Toothman & Barrett, 2011). Interestingly, the second trend seems to be driven by men’s ratings (Billari et al., 2021), as women raters do perceive men and women to enter old age at very similar ages. As discussed before, women might set the onset of old age later than men because their average life expectancy is longer than that of men (German Federal Statistical Office, n.d.), but they might also be influenced by the “double standard of aging” (Sontag, 1982), which discriminates older women and therefore set an older onset of old age as a means of “age-group dissociation” (Weiss & Lang, 2012). The double standard of aging might also explain why men in particular perceive women to enter old age earlier than men. This gender difference might additionally be due to the fact that women, while living longer than men, are more affected by multimorbidity (Fuchs et al., 2012) than men (“women suffer but men die”; Phillips et al., 2023). Thus, when comparing women and men in old and very old age, there will be a greater selectivity of healthy survivors among men than women.

Some of the macro-level factors revealed a significant interaction with the raters’ gender. For instance, the finding that a steeper increase in women’s statutory retirement age over time was related to a less pronounced increase in the perceived onset of old age was restricted to male raters. This finding can be taken to suggest that men and women not only differ in their perceived onset of old age and in when they are considered as “old,” they also differ in how macro-level indicators are associated with their perceived onset of old age.

Additionally, the role of the macro-level factors varied by “gender target.” For instance, in countries with a later statutory retirement age for men, the change toward a later perceived onset of male targets was more pronounced than in countries with an earlier male statutory retirement age; however, the change in the perceived onset of old age of female targets was not related to the male statutory retirement age. Gender-specific ratings of the onset of old age thus seem to be associated with gender-specific macro-level indicators.

### Study limitations

Some cautions in interpretation and generalization are warranted. Regarding the study design, we cannot draw causal conclusions from this observational study and from the associations we investigated. It is, for instance, possible that perceptions of old age in a society influence how the retirement age is set or that perceptions of old age have motivational and behavioral consequences that translate into a longer, healthy life expectancy.

Moreover, we took gender differentiation into account for one of the macro-level indicators, namely retirement age. Future research should also consider gender differences in other macro-level indicators, such as healthy life expectancy or older adults’ employment rates, and the implications of such gender differences for the perceived onset of old age. Future research should also widen the range of macro-level indicators.

Finally, analyses of historical change require that the samples recruited in different decades are comparable. Given the general trend toward declining survey participation (Luiten et al., 2020), this is, however, a challenge, and the samples drawn in 2018 might be more positively selected than those drawn in 2006. While analysis with sample weights reduces such selection biases, the extent of historical increase in the perceived onset of old age may still be somewhat overestimated.

## Conclusions

Our results indicate that within 12 years, the perceived onset of old age has been “postponed” by more than 3 years. Moreover, our findings suggest that gender is an important factor to consider in the context of perceived onset of old age, and gender needs to be taken into account both from the rater’s perspective and from the target’s perspective. Female raters report a later perceived onset of old age than male raters, and women as targets are perceived to enter old age earlier than men as targets.

Finally, our findings suggest that variations in macro-level contexts and their changes across countries are related to cross-country variations in the perceived onset of old age and its temporal trend over 12 years, and some of these associations between macro-level indicators and the perceived onset of old age were additionally moderated by gender. Our findings thus suggest that the perceived onset of old age is not only shaped by an individual’s age or gender but also by societal, macro-level factors and how they change over historical time. Promoting positive views on aging might thus require “macro-level efforts,” in addition to interventions and measures on the micro-level.

From a theoretical point of view, frameworks on views on aging, including the perceived onset of old age, or on historical change in development, need to take into account that such historical changes are not only shaped by macro-level and micro-level factors but also by their interactions, as, for instance, illustrated by our findings of gender-specific effects of certain macro-level factors.

## Author notes

1. Although the effective and statutory retirement age are quite discrepant in many European countries such as Germany (Demografieportal, 2025; Weber & Loichinger 2022), one can assume that the statutory retirement age is more salient to people due to its prominence in political and societal discussion and thus a more important “anchor” for the perceived onset of old age, whereas not everybody is aware of the average effective retirement age in their country.
2. To check whether our comprehensive set of country-level predictors and their interaction terms could be accommodated by the macro-level sample size, we compared these full models with reduced models that do not include these interactions. The full models provided a significantly better fit without convergence issues (All targets: χ^2^(28) = 254.73, *p* < .001; Men targets: χ^2^(28) = 174.47, *p* < .001; Women targets: χ^2^(28) = 136.20, *p* < .001).
3. To compare results across different weighting methods, we additionally computed sensitivity analyses by fitting unweighted and weighted mixed models based on the rescaled weights (Carle, 2009; Wettstein et al., 2024) using the R “WeMix” package (version 4.0.3); results are presented in the supplementary material. As recommended in the ESS guidelines, analysis weights were used in the main analysis—as well as in the mean-centering—to adjust for differential sampling probabilities, sampling, nonresponse, or noncoverage errors, and age, gender, and regional differences in population size across countries (Kaminska & Lynn, 2017); post-stratification weights that adjust for age, gender, and regional differences in population size across countries were used for descriptive analyses (Billari et al., 2021).

## Supplementary Material

gbag083_Supplementary_Data

## Data Availability

Data were drawn from the European Social Survey, which are available for researchers (https://ess.sikt.no/en/data-builder/). Additionally, we used data from the OECD Data Explorer (accessible at: https://data-explorer.oecd.org/), the Social Security Administration (accessible at: https://www.ssa.gov/policy/docs/progdesc/ssptw/2006-2007/europe/ssptw06euro.pdf), and the Eurostat database (accessible at: https://ec.europa.eu/eurostat/databrowser/view/TEPSR_SP320__custom_18030940/default/table). Study materials are available at: https://www.europeansocialsurvey.org/methodology/ess-methodology/data-and-documentation-availability. This study was not preregistered. This contribution is based on a secondary data analysis; we cannot make the data from the European Social Survey available, but they can be accessed as described in the data availability section. The analytical code is provided in the Supplementary Material.
