# Supplementary material for: Old age is perceived to begin later: cross-European differences and the role of macro-level factors for the historical change in the perceived onset of old age
Source: J Gerontol B Psychol Sci Soc Sci. 2026 May 12;81(7):gbag083. doi: 10.1093/geronb/gbag083 (PMC13275313; doi:10.1093/geronb/gbag083)
Supplement: gbag083_Supplementary_Data [file gbag083_supplementary_data.docx]

***The Journals of Gerontology, Series B: Psychological Sciences and Social Sciences* Supplementary Material: Wettstein et al. Old age is perceived to begin later: Cross-European differences and the role of macro-level factors for historical change in perceived onset of old age. Explanation of Model Equations**

Interpretation of parameters associated with country-level variables (e.g., employment rate) is as follows. In Eq. 2.1, *γ_00_* is the expected onset of old age for male raters in 2006 in the average country, *γ_01_* is the difference in onset of old age for male raters in 2006 for a one unit higher (i.e., 10%) initial employment rate (*Employ_j_* = employment rate in 2006), and *u_0j_* is the country-specific random effect that is assumed to follow a normal distribution *N*(0, *σ_u_*^2^); in Eq. 2.2., *γ_10_* is the expected amount of change in onset of old age for male raters (2018 - 2006) in average country, *γ_11_* is the difference in change in onset of old age for a one unit higher (i.e., 10%) initial employment rate, *γ_12_* is the difference in change in onset of old age for a one unit increase (i.e., 1%) in the change in employment rate (change-on-change association), and *γ_13_* is the extent to which the change-on-change association is moderated by initial employment rate; in Eq. 2.3, *γ_20_* is the gendered rater bias in perceived onset of old age in 2006 in average country and *γ_21_* is the how the gender bias in onset in 2006 is related to country-level differences in initial employment rate; in Eq. 2.4, *γ_30_* is the change in gender bias in onset (2018 – 2006) in average country, *γ_31_* is the how the gender bias change is related to country-level differences in initial employment rate, *γ_32_* is the how the gender bias change is related to country-level differences in the change in employment rate, and *γ_33_* is the extent to which that association is moderated by initial employment rate. Finally, for those parameters that depend on person-level variables only, *γ_40_* is the prototypical difference in onset for male raters in 2006 for a unit higher (i.e., 10 years) age (*Age_ij_*) in Eq. 2.5, and in Eq. 2.6, *γ_50_* is the prototypical difference in onset for a unit higher (i.e., 10 years) education level (*Education_ij_*). Taken together, the model accounts for macro-level (parameters in Eq. 2.1 to Eq. 2.4) and micro-level (parameters in Eq. 2.5 to Eq. 2.6) differences in the perceived onset of old age across the two years.

**Table S1**

*Country-Level Descriptive Statistics of Macro-Level Factors and Perceived Onset of Old Age*

| **Country** | | **Year** | **Employ** | **Men**  **Retire** | **Women**  **Retire** | **HLE** |
| --- | --- | --- | --- | --- | --- | --- |
| Austria | (AT) | 2006 | 33.0 | 65.0 | 60.0 | 7.4 |
|  |  | 2018 | 54.0 | 65.0 | 60.0 | 7.4 |
| Belgium | (BE) | 2006 | 32.0 | 65.0 | 63.0 | 9.9 |
|  |  | 2018 | 50.4 | 65.0 | 65.0 | 11.1 |
| Germany | (DE) | 2006 | 48.1 | 65.0 | 65.0 | 7.6 |
|  |  | 2018 | 70.4 | 65.5 | 65.5 | 11.9 |
| Denmark | (DK) | 2006 | 60.7 | 65.0 | 65.0 | 13.4 |
|  |  | 2018 | 69.2 | 65.0 | 65.0 | 11.3 |
| Estonia | (EE) | 2006 | 58.4 | 63.0 | 63.0 | 4.0 |
|  |  | 2018 | 68.4 | 63.3 | 63.3 | 5.7 |
| Spain | (ES) | 2006 | 44.1 | 65.0 | 65.0 | 9.8 |
|  |  | 2018 | 52.1 | 65.0 | 65.0 | 11.4 |
| Finland | (FI) | 2006 | 54.5 | 65.0 | 65.0 | 6.9 |
|  |  | 2018 | 64.6 | 65.0 | 65.0 | 9.5 |
| France | (FR) | 2006 | 39.4 | 60.0 | 60.0 | 9.2 |
|  |  | 2018 | 53.6 | 63.3 | 63.3 | 10.8 |
| United Kingdom | (UK) | 2006 | 57.3 | 65.0 | 60.0 | 10.7 |
|  |  | 2018 | 65.3 | 65.0 | 62.7 | 10.4 |
| Hungary | (HU) | 2006 | 33.2 | 62.0 | 62.0 | 5.4 |
|  |  | 2018 | 54.4 | 63.5 | 62.0 | 7.2 |
| Ireland | (IE) | 2006 | 53.1 | 65.0 | 65.0 | 9.8 |
|  |  | 2018 | 60.2 | 66.0 | 66.0 | 12.9 |
| Netherlands | (NL) | 2006 | 44.0 | 65.0 | 65.0 | 11.2 |
|  |  | 2018 | 67.5 | 65.8 | 65.8 | 9.7 |
| Norway | (NO) | 2006 | 67.4 | 67.0 | 67.0 | 12.0 |
|  |  | 2018 | 72.0 | 67.0 | 67.0 | 15.4 |
| Poland | (PL) | 2006 | 28.1 | 65.0 | 60.0 | 7.8 |
|  |  | 2018 | 48.4 | 65.0 | 60.8 | 8.5 |
| Portugal | (PT) | 2006 | 50.0 | 65.0 | 65.0 | 6.5 |
|  |  | 2018 | 57.2 | 65.2 | 65.2 | 7.3 |
| Slovenia | (SI) | 2006 | 32.6 | 61.8 | 55.4 | 9.0 |
|  |  | 2018 | 46.6 | 62.0 | 61.7 | 7.4 |
| Slovakia | (SK) | 2006 | 33.0 | 62.0 | 61.7 | 3.9 |
|  |  | 2018 | 56.0 | 62.0 | 62.0 | 4.4 |

Note: *N* = 55,721 from 17 countries. Average ratings of perceived onset of old age for all targets/men targets/women targets for each country in each year of assessment are given (sample sizes in parentheses). Employ = employment rates of adults aged 55-64 years. Men Retire = men’s statutory retirement age. Women Retire = women’s statutory retirement age. HLE = healthy life expectancy at age 65.

Post-stratification weights have been applied for country-level analysis. Observed changes in 2018 (vs. 2006) are highlighted in yellow (positive) or blue (negative).

**Table S2**

*Country-Level Descriptive Statistics (with Post-Stratification Weights)*

| **Country** | **Year** | **Female Ratio** | **Age** | **Education** | **Perceived Onset of Old Age^#^** | | |
| --- | --- | --- | --- | --- | --- | --- | --- |
|  |  |  |  |  | **All**  **Targets** | **Men**  **Targets** | **Women**  **Targets** |
| Austria | 2006 | 0.51 | 45.5 | 12.6 | 68.4 | 67.6 | 69.1 |
|  | 2018 | 0.52 | 48.3 | 13.3 | 71.2 | 72.4 | 70.1 |
| Belgium | 2006 | 0.51 | 45.3 | 11.7 | 68.4 | 68.9 | 67.8 |
|  | 2018 | 0.51 | 47.2 | 13.6 | 69.7 | 69.8 | 69.6 |
| Germany | 2006 | 0.51 | 46.7 | 12.7 | 65.2 | 66.1 | 64.3 |
|  | 2018 | 0.50 | 47.6 | 13.6 | 65.1 | 65.2 | 64.9 |
| Denmark | 2006 | 0.51 | 46.9 | 12.8 | 72.2 | 73.5 | 70.7 |
|  | 2018 | 0.51 | 46.7 | 13.3 | 71.0 | 71.8 | 70.1 |
| Estonia | 2006 | 0.56 | 45.2 | 12.3 | 64.4 | 64.7 | 64.1 |
|  | 2018 | 0.54 | 47.7 | 13.7 | 66.6 | 66.0 | 67.2 |
| Spain | 2006 | 0.51 | 46.0 | 11.9 | 66.6 | 67.2 | 66.1 |
|  | 2018 | 0.52 | 48.2 | 13.5 | 67.7 | 68.0 | 67.4 |
| Finland | 2006 | 0.52 | 46.0 | 12.6 | 67.6 | 67.1 | 68.2 |
|  | 2018 | 0.51 | 48.3 | 13.7 | 68.4 | 68.2 | 68.6 |
| France | 2006 | 0.52 | 45.6 | 12.1 | 69.5 | 70.9 | 68.1 |
|  | 2018 | 0.53 | 47.6 | 12.9 | 70.1 | 72.5 | 67.7 |
| UK | 2006 | 0.64 | 56.7 | 16.9 | 85.2 | 85.3 | 85.1 |
|  | 2018 | 0.51 | 47.4 | 14.5 | 69.7 | 70.2 | 69.2 |
| Hungary | 2006 | 0.58 | 48.9 | 12.8 | 66.0 | 66.1 | 65.9 |
|  | 2018 | 0.52 | 48.4 | 12.3 | 63.7 | 64.3 | 63.2 |
| Ireland | 2006 | 0.60 | 44.0 | 13.4 | 72.0 | 72.1 | 71.9 |
|  | 2018 | 0.52 | 45.7 | 15.0 | 71.6 | 68.8 | 74.3 |
| Netherlands | 2006 | 0.51 | 46.0 | 13.6 | 71.6 | 71.3 | 71.9 |
|  | 2018 | 0.50 | 46.5 | 13.9 | 71.4 | 71.1 | 71.7 |
| Norway | 2006 | 0.51 | 45.3 | 12.7 | 69.6 | 70.7 | 68.6 |
|  | 2018 | 0.48 | 44.8 | 13.2 | 69.1 | 68.7 | 69.5 |
| Poland | 2006 | 0.52 | 43.3 | 11.4 | 62.4 | 63.1 | 61.8 |
|  | 2018 | 0.52 | 47.6 | 12.8 | 66.1 | 66.7 | 65.5 |
| Portugal | 2006 | 0.52 | 46.4 | 7.6 | 69.4 | 67.7 | 70.9 |
|  | 2018 | 0.53 | 48.3 | 10.0 | 71.3 | 71.5 | 71.1 |
| Slovenia | 2006 | 0.51 | 45.0 | 11.6 | 64.1 | 64.2 | 64.1 |
|  | 2018 | 0.50 | 48.6 | 12.6 | 65.3 | 65.5 | 65.1 |
| Slovakia | 2006 | 0.55 | 42.9 | 12.3 | 64.3 | 65.4 | 63.2 |
|  | 2018 | 0.52 | 46.0 | 12.6 | 64.1 | 66.5 | 61.7 |

Note: UK = United Kingdom. Post-stratification weights that account for differences in age group, gender, education, and region have been applied.

**Table S3**

*Inclusion of Survey Weights in Multilevel Regression on Perceived Onset of Old Age.*

|  | **With covariate** | | | | |
| --- | --- | --- | --- | --- | --- |
|  | **Unweighted** | **Combined** | **Level-1** | **Level-2** | **Separate** |
| ***Fixed effects*** |  |  |  |  |  |
| **Intercept (*γ_00_*)** | **66.46*** (1.24)** | **66.64*** (1.25)** | **66.46*** (1.23)** | **66.58*** (1.26)** | **64.40*** (0.70)** |
| Employ (*γ_01_*) | 0.15 (0.60) | 0.21 (0.61) | 0.14 (0.59) | 0.23 (0.62) | 0.59 (0.40) |
| MenRetire (*γ_02_*) | -1.11 (4.28) | -1.32 (4.29) | -1.36 (4.24) | -0.95 (4.34) | -6.33*** (1.40) |
| WomenRetire (*γ_03_*) | 2.90 (2.71) | 3.42 (2.75) | 3.06 (2.68) | 3.01 (2.78) | 2.63 (1.33) |
| **Healthy (*γ_04_*)** | **5.81* (2.36)** | **5.86* (2.42)** | **5.81* (2.34)** | **5.90* (2.44)** | **8.21** (2.70)** |
| **Year (*γ_10_*)** | **3.88*** (0.70)** | **3.70*** (0.91)** | **3.72*** (0.64)** | **3.80*** (1.08)** | **3.73*** (0.36)** |
| Year × Employ (*γ_11_*) | -0.09 (0.32) | -0.33 (0.40) | -0.00 (0.32) | -0.43 (0.41) | -0.30 (0.16) |
| **Year × MenRetire (*γ_12_*)** | **7.56*** (2.09)** | **5.58* (2.74)** | **5.47** (1.97)** | **7.49* (3.07)** | **5.60*** (1.12)** |
| Year × WomenRetire (*γ_13_*) | -2.74** (0.91) | -3.01* (1.23) | -1.71 (0.88) | -3.67** (1.29) | -2.84*** (0.89) |
| Year × Healthy (*γ_14_*) | -2.18*  (0.93) | -1.83 (1.30) | -2.41** (0.88) | -1.42 (1.47) | -1.98*** (0.51) |
| **Year × ChangeEmploy (*γ_15_*)** | **-0.12*** (0.03)** | **-0.14*** (0.04)** | **-0.13*** (0.03)** | **-0.13** (0.04)** | **-0.14*** (0.03)** |
| **Year × ChangeMenRetire (*γ_16_*)** | **2.32*** (0.46)** | **2.05* (0.87)** | **1.68*** (0.45)** | **2.62** (0.83)** | **1.98*** (0.62)** |
| Year × ChangeWomenRetire (*γ_17_*) | -0.45*  (0.21) | -0.59*  (0.30) | -0.39 (0.21) | -0.67* (0.29) | -0.57** (0.20) |
| Year × ChangeHealthy (*γ_18_*) | -0.20*  (0.10) | -0.23*  (0.12) | -0.28** (0.09) | -0.15 (0.13) | -0.25*** (0.07) |
| Year × Employ × ChangeEmploy (*γ_19_*) | -0.04 (0.03) | -0.04 (0.03) | -0.05* (0.03) | -0.03 (0.03) | -0.04 (0.02) |
| Year × MenRetire × ChangeMenRetire (*γ_110_*) | 1.83* (0.73) | 1.92 (1.33) | 1.37 (0.72) | 2.29 (1.29) | 1.82 (0.96) |
| Year × WomenRetire × ChangeWomenRetire (*γ_111_*) | -0.22 (0.22) | -0.19 (0.41) | -0.12 (0.21) | -0.33 (0.42) | -0.21 (0.17) |
| Year × Healthy × ChangeHealthy (*γ_112_*) | -0.70 (0.38) | -0.60 (0.57) | -0.64 (0.38) | -0.70 (0.57) | -0.62 (0.38) |
| **FemaleRater (*γ_20_*)** | **2.35*** (0.21)** | **2.09*** (0.20)** | **2.48*** (0.22)** | **2.12*** (0.19)** | **2.10*** (0.21)** |
| FemaleRater × Employ (*γ_21_*) | 0.22* (0.11) | 0.19 (0.12) | 0.24* (0.11) | 0.14 (0.12) | 0.19 (0.14) |
| FemaleRater × MenRetire (*γ_22_*) | -0.24 (0.73) | -0.23 (0.60) | 0.30 (0.75) | -0.72 (0.57) | -0.22 (0.43) |
| FemaleRater × WomenRetire (*γ_23_*) | -0.01 (0.47) | -0.86 (0.46) | -0.14 (0.48) | -0.36 (0.45) | -0.86 (0.55) |
| FemaleRater × Healthy (*γ_24_*) | 1.37** (0.43) | 0.50 (0.68) | 0.99* (0.44) | 0.74 (0.64) | 0.49 (0.83) |
| Year × FemaleRater (*γ_30_*) | 0.41 (0.84) | -0.08 (1.05) | -0.19 (0.74) | 0.54 (1.29) | -0.06 (0.41) |
| Year × FemaleRater × Employ (*γ_31_*) | 0.24 (0.39) | 0.80 (0.47) | 0.42 (0.37) | 0.69 (0.50) | 0.79*** (0.23) |
| Year × FemaleRater × MenRetire (*γ_32_*) | 4.24 (2.54) | 4.59 (3.15) | 3.64 (2.32) | 5.69 (3.67) | 4.71*** (1.44) |
| Year × FemaleRater × WomenRetire (*γ_33_*) | 1.16 (1.12) | 2.24 (1.45) | 0.37 (1.08) | 2.55 (1.54) | 2.20 (1.10) |
| Year × FemaleRater × Healthy (*γ_34_*) | -0.68 (1.14) | -0.24 (1.58) | 0.12 (1.06) | -1.21 (1.80) | -0.20 (0.82) |
| Year × FemaleRater × ChangeEmploy (*γ_35_*) | 0.00 (0.03) | 0.03 (0.05) | 0.00 (0.03) | 0.03 (0.05) | 0.03 (0.04) |
| Year × FemaleRater × ChangeMenRetire (*γ_36_*) | -1.19* (0.51) | -1.21 (0.98) | -0.61 (0.51) | -1.74 (0.93) | 1.15 (0.93) |
| **Year × FemaleRater × ChangeWomenRetire (*γ_37_*)** | **0.51* (0.24)** | **0.71* (0.34)** | **0.53* (0.23)** | **0.71* (0.34)** | **0.71*** (0.22)** |
| Year × FemaleRater × ChangeHealthy (*γ_38_*) | 0.23* (0.11) | 0.29* (0.14) | 0.34** (0.11) | 0.18 (0.15) | 0.29*** (0.09) |
| Year × FemaleRater × Employ × ChangeEmploy (*γ_39_*) | -0.02 (0.03) | -0.04 (0.04) | -0.02 (0.03) | -0.03 (0.04) | -0.04 (0.02) |
| Year × FemaleRater × MenRetire × ChangeMenRetire (*γ_310_*) | -2.43** (0.81) | -2.58 (1.50) | -1.51 (0.81) | -3.53*  (1.44) | -2.52 (1.42) |
| Year × FemaleRater × WomenRetire × ChangeWomenRetire (*γ_311_*) | -0.10 (0.25) | -0.04 (0.47) | -0.05 (0.25) | -0.08 (0.48) | -0.04 (0.21) |
| Year × FemaleRater × Healthy × ChangeHealthy (*γ_312_*) | -0.96*  (0.44) | -1.01  (0.65) | -0.97*  (0.43) | -1.06 (0.67) | -0.99 (0.48) |
| **Age (*β_4_*)** | **1.15*** (0.02)** | **1.32*** (0.02)** | **1.23*** (0.02)** | **1.25*** (0.02)** | **1.33*** (0.15)** |
| **Education (*β_5_*)** | **1.48*** (0.08)** | **2.11*** (0.08)** | **1.56*** (0.08)** | **2.05*** (0.08)** | **2.13*** (0.25)** |
| ***Random effects*** |  |  |  |  |  |
| Std. Dev. Intercept (*σ_u0_*) | 2.15 | 2.15 | 2.12 | 2.18 | 1.45 |
| Residual Std. Dev. (*σ_e_*) | 7.78 | 8.17 | 7.87 | 7.66 | 7.98 |
| ***Goodness-of-fit statistics*** |  |  |  |  |  |
| Log-likelihood (*df*) | -193470 (40) | -213808 (40) | -197052 (40) | -209435 (40) | -184840 (40) |
| AIC | 387021 | 427696 | 394184 | 418951 | 369760 |

Note: *N* = 55,721 from 17 countries. Regression coefficients with standard errors in parentheses. Models fitted with **Level-2** country-level weights *w_j_*^(2)^ *= w_j,2006_* measured in 2006, **Level-1** person-level weights *w_i|j_*^(1)^ that are conditioned on the Level-2 weights and rescaled with Method A (Carle, 2009), both Level-1 and Level-2 weights applied **separately** at each level, or in a **combined** way *w_ij_* = *w_j_*^(2)^ × *w_i|j_*^(1)^, using the R packages “lme4” (version 1.1-35) [Unweighted, Combined, Level-1, Level-2] and “WeMix” (version 4.0.3) [Separate]. SE = standard error. Std. Dev. = standard deviation. ****p* < .001, ** *p* < .01, **p* < .05.

**Figure S1**

*Country-Level Predictions for Change in Perceived Onset of Old Age in Relation to Healthy Life Expectancy.*

**
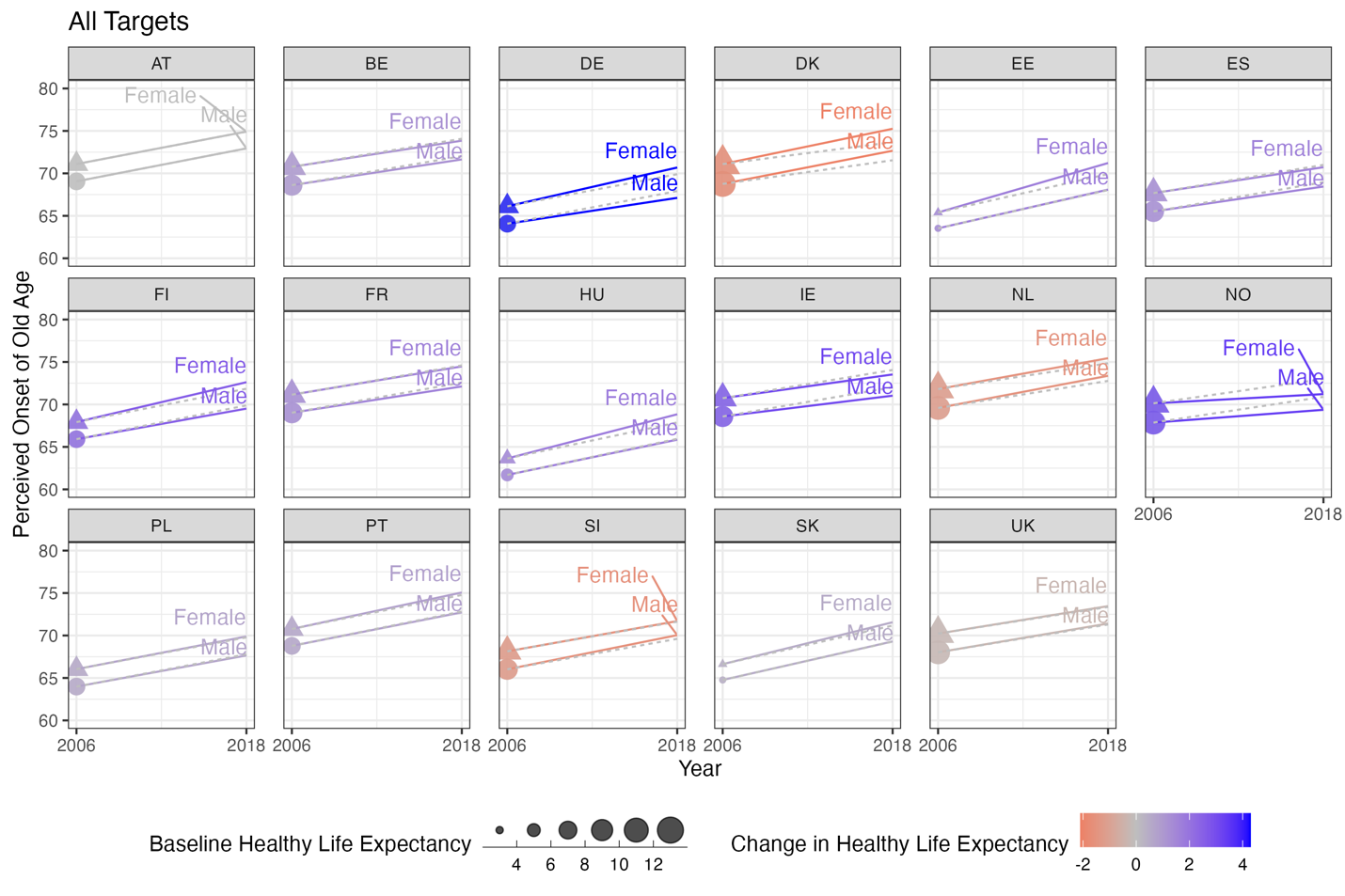
**

Note: Model predictions for male (circle) and female (triangle) raters in each country, given healthy life expectancy in 2006 and the observed amount of change in healthy life expectancy between 2006 and 2018 (solid line); dotted lines: model predictions without the change in healthy life expectancy across two cohorts. AT = Austria; BE = Belgium; DE = Germany; DK = Denmark; EE = Estonia; ES = Spain; FI = Finland; FR = France; HU = Hungary; IE = Ireland; NL = Netherlands; NO = Norway; PL = Poland; PT = Portugal; SI = Slovenia; SK = Slovakia; UK = United Kingdom.

**Figure S2** *Country-Level Predictions for Change in Perceived Onset of Old Age in Relation to Healthy Life Expectancy, for Men as Targets vs. Women as Targets*

**Figure S2A**

**
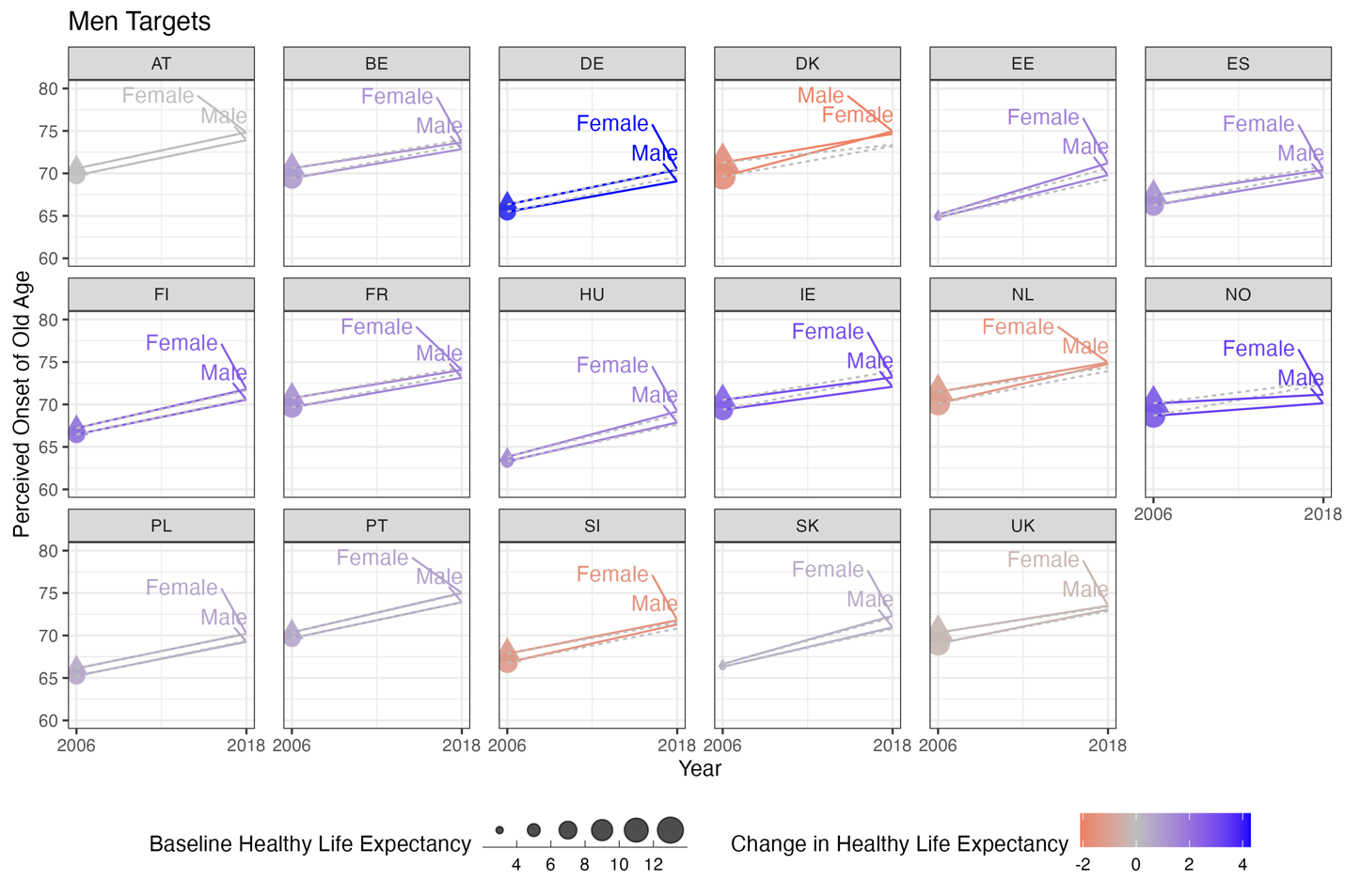
**

Note: “Male” (circle) and “female” (triangle) refers to the gender of the raters. AT = Austria; BE = Belgium; DE = Germany; DK = Denmark; EE = Estonia; ES = Spain; FI = Finland; FR = France; HU = Hungary; IE = Ireland; NL = Netherlands; NO = Norway; PL = Poland; PT = Portugal; SI = Slovenia; SK = Slovakia; UK = United Kingdom.

**Figure S2B**


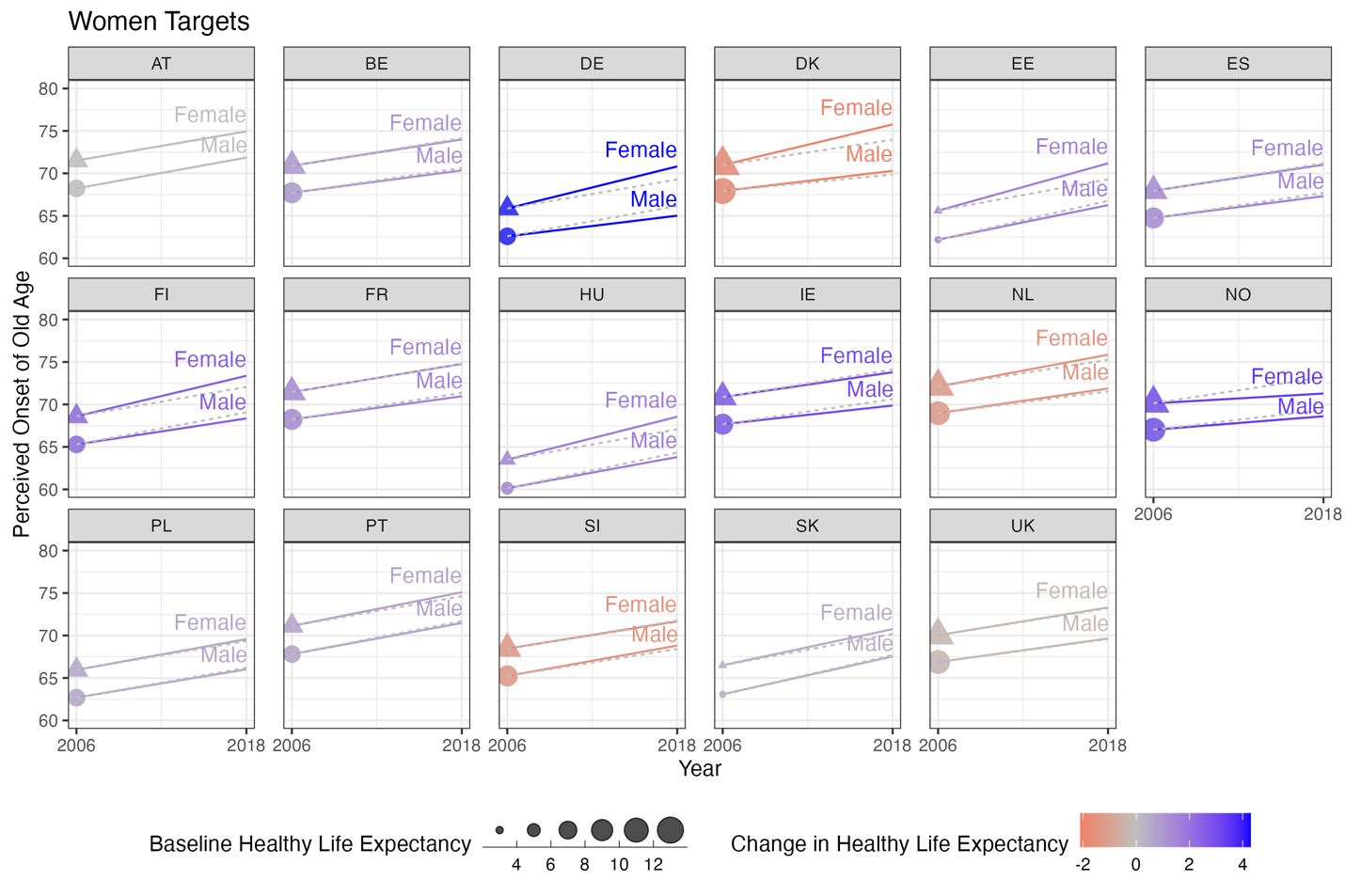


Note: “Male” (circle) and “female” (triangle) refers to the gender of the raters AT = Austria; BE = Belgium; DE = Germany; DK = Denmark; EE = Estonia; ES = Spain; FI = Finland; FR = France; HU = Hungary; IE = Ireland; NL = Netherlands; NO = Norway; PL = Poland; PT = Portugal; SI = Slovenia; SK = Slovakia; UK = United Kingdom.

**Analytical Code for R:**

# Multilevel Modeling

```{r}

# Define syntax for modeling onset of old age

mod_syntax <- formula(

ageoage ~

1+emplB+mrtrB+wrtrB+hleB+ #b0

cohort+cohort:(emplB+mrtrB+wrtrB+hleB+

emplD+mrtrD+wrtrD+hleD+

emplB:emplD+mrtrB:mrtrD+wrtrB:wrtrD+hleB:hleD)+ #b1

female+female:(emplB+mrtrB+wrtrB+hleB)+ #b2

cohort:female+cohort:female:(emplB+mrtrB+wrtrB+hleB+

emplD+mrtrD+wrtrD+hleD+

emplB:emplD+mrtrB:mrtrD+wrtrB:wrtrD+hleB:hleD)+ #b3

agea+education+ #b4

(1|cntry)

)

```

```{r}

## Column 1: All targets

lmer_mod <- lmer(

mod_syntax,

control = lmerControl(optimizer="bobyqa",calc.derivs=FALSE),

weights = anweight,

data = mydata

)

summary(lmer_mod)

```

Split-ballot design - men targets.

```{r}

## Column 2: Men targets

lmer_modM <- lmer(

mod_syntax,

control = lmerControl(optimizer="bobyqa",calc.derivs=FALSE),

weights = anweight,

data = mydata %>% filter(splitcode=="Men")

)

summary(lmer_modM)

```

Split-ballot design - women targets.

```{r}

## Column 3: Women targets

lmer_modW <- lmer(

mod_syntax,

control = lmerControl(optimizer="bobyqa",calc.derivs=FALSE),

weights = anweight,

data = mydata %>% filter(splitcode=="Women")

)

summary(lmer_modW)

```

# R Environment

```{r}

sessionInfo()

```
